# Efficacy of guided self-change for smoking cessation in chronic obstructive pulmonary disease patients: A randomized controlled clinical trial

**DOI:** 10.18332/tid/114227

**Published:** 2019-12-11

**Authors:** Mehran Zarghami, Fatemeh Taghizadeh, Ali Sharifpour, Abbas Alipour

**Affiliations:** 1 Department of Psychiatry, School of Medicine, Mazandaran University of Medical Sciences, Sari, Iran; 2 Psychiatry and Behavioral Sciences Research Center, Addiction Institute, Mazandaran University of Medical Sciences, Sari, Iran; 3 Pulmonary and Critical Care Division, Iranian National Registry Center for Lophomoniasis, Mazandaran University of Medical Sciences, Sari, Iran; 4 Toxoplasmosis Research Center, Iranian National Registry Center for Lophomoniasis, Mazandaran University of Medical Sciences, Sari, Iran; 5 Department of Epidemiology, Mazandaran University of Medical Sciences, Sari, Iran

**Keywords:** quality of life, smoking cessation, chronic obstructive pulmonary disease

## Abstract

**INTRODUCTION:**

The aim of this study was to examine the efficacy of guided self-change (GSC), nicotine replacement therapy (NRT), and their combination, on smoking cessation among patients with COPD.

**METHODS:**

A total of 60 participants were randomly assigned to three groups for GSC (n=20), nicotine replacement therapy (NRT) (n=20) or their combination (n=20), from December 2016 to November 2017. The quality of life (QoL) questionnaire, clinical assessment test (CAT) and exhaled carbon monoxide (CO), were measured at baseline and post-treatment.

**RESULTS:**

At 6, 12, and 29 weeks, the abstinence rate in the NRT group was 5.3%, 15.8% and 21.1%, in the GSC group 21.1%, 31.6% and 47.4%, and in the combined group 36.8%, 36.8% and 47.4%, respectively. The exhaled CO in the NRT group was greater than the GSC group, however this difference was not statistically significant (3.4; 95% CI: -0.24–7.0; p=0.067), CO levels in the combined group were less than the GSC group, while this difference was also not significant (-0.75; 95% CI : -4.2–2.7; p=0.68). CAT and QoL recovery in the GSC and combined groups were higher than in the NRT group (9.2; 95% CI: 5.0–13.4; p=0.001) and (-4.5; 95% C: -8.1– -0.6; p=0.02), respectively. However, differences between combined and GSC groups were not significant (p=0.24 and p=0.41, respectively). There was a statistically significant difference between the abstinence rate in the GSC or combined group and the NRT group (p=0.001). The GEE model showed that GSC reduced the odds of smoking compared with the NRT group (interaction group effect) (OR=0.31, 95% CI: 0.022–0.545; p=0.001).

**CONCLUSIONS:**

In our context among COPD patients, GSC was more effective in decreasing smoking than NRT alone. Moreover, the recovery of exhaled carbon monoxide, CAT and QoL in GSC was more than in the NRT group. Moreover, since GSC was as effective as GSC plus NRT, the effectiveness of the combination method for smoking cessation in COPD patients may be attributed to GSC.

Clinical trial registration details: IRCT201609271457N11; www.irct.ir

## INTRODUCTION

Tobacco smoking is as a major public health issue^1^ and also an important risk factor for various diseases, such as chronic obstructive pulmonary disease (COPD)^2^. Smoking is the most common causative factor for COPD and about half of all smokers develop this condition at older ages^3^. Smoking is also a common risk factor for disease development as a mean smoking prevalence of 22.2% was obtained for 126 countries^4^. Smoking rates have been estimated at 19.2% in Northern Iranian male subjects with COPD^5^.

Smoking is a global health crisis, which decreases a patient’s quality of life (QoL) and pulmonary function^6^. Smoking cessation is recommended as the most effective approach to increase pulmonary function and improve the respiratory symptoms in COPD patients^7^. Various pharmaceutical methods such as varenicline, bupropion and nicotine replacement therapy have been used to stop smoking. Due to the side effects of varenicline and bupropion, many patients prefer nicotine replacement therapy (NRT)^8,9^. The reasons for considering NRT in this study are its low side effects and popularity among patients. Prescription of systemic nicotine is a medically-approved medication that supplies low doses of nicotine without coal tar and carbon monoxide — which are major risk factors for pulmonary diseases — and increases the chance of smoking cessation and the chances of quitting smoking by about 55%^10^, and is more effective if combined with behavioral treatments^11^ such as guided self-change (GSC). GSC has been influenced by three major domains: Brief Intervention, Natural Recovery, and Motivational Intervention^12^. Patients undergoing GSC allocate less time to learn and train than those undergoing cognitive-behavioural therapy (CBT) when they are presented with their self-treatment manual. As we are unable to identify a related study to assess the impact of GSC on smoking cessation in COPD patients, this study aimed to examine the efficacy of GSC for smoking cessation in COPD patients in a randomized controlled trial.

## METHODS

In a Randomized Controlled Clinical Trial (IRCT registration number: IRCT201609271457N11), 60 eligible COPD patients in the Imam Khomeini hospital (Sari, Mazandaran, Iran) were randomly assigned to a group for GSC (n=20), NRT (n=20) or for their combination (n=20) to study the quitting rate, in accordance with the Declaration of Helsinki and its subsequent revisions, from December 2016 to November 2017. NRT was included as a control group as a known effective method, and the hypothesis of comparing the GSC with GSC + NRT was to study the probable additional effect of a combination of the new method and the known effective method.

### Inclusion and exclusion criteria

Inclusion criteria were: age over 45 years, participation in at least four treatment sessions, with current COPD and nicotine dependence diagnosed and referred by a pulmonologist. In this study, only men were recruited.

Exclusion criteria included: younger than 45 years with other systemic diseases such as diabetes mellitus, respiratory failure, normal primary spirometry, contraindications for nicotine gum (allergy, recent heart attacks, dangerous arrhythmias, severe angina, hyperthyroidism, insulin-dependent diabetes mellitus, active peptic ulcers, pregnancy and lactation) or a history of severe psychiatric disorders including psychosis, severe depression and anxiety in the patient’s medical history with GSC psychotherapist and psychiatrist diagnosis.

### Randomization, concealment and blinding

The researchers first performed the baseline assessments. Then the envelopes were sealed and numbered. Next, the opaque envelopes containing allocation codes were opened by the research assistant. The allocation codes were produced using a computerized block randomization program by an independent clinical epidemiologist, who was not involved in the recruitment, intervention or the clinical assessment. The participants who met the inclusion criteria were randomized into three equal groups, according to the randomization list, after signing the informed consent form. The participants and therapist were blind to the allocation; however, neither participants nor the therapist was blinded during the clinical trial sessions.

### Procedures and measurements

All randomized participants were referred to the Mostafavian Pulmonary Clinic of Imam Khomeini Hospital in Sari, located in northern Mazandaran province, Iran. The present study was approved by the Ethics Committee of the hospital and all of the patients provided a written informed consent.

After randomization, further information including educational level, medical history, smoking, quitting history, and other related data, were collected. A sample size of 60 patients was calculated (conferring 80% power and 5% significance with two-sided tests in order to detect an absolute difference of 10% in quitting rates across the three groups) with 20 participants considered for each group. After explaining the study protocol, the patients completed the questionnaires, including demographic information, the clinical assessment test (CAT), Fagerström test for nicotine dependence (FTND)^13^ and quality of life (QoL), at baseline and also at 12 and 29 weeks after treatment. The SF-12 is a reliable and valid measure of health-related quality of life among Iranians^14^. Self-reported smoking was recorded ten times during the study and verified by exhaled carbon monoxide level (Bedfont PiCO + Smokerlyzers, Bedfont Scientific, UK)^15^.

Forced expiratory volume in one second (FEV1) and forced vital capacity (FVC) were measured before the intervention and during the treatment, to assess any further decline/improvement of lung function relative to the baseline, using a spirometry device. The normal spirometry results were defined as FEV1/FVC ≥70% and FVC ≥80%^16^. The spirometry test was repeated every six weeks after treatment. Besides usual treatments (bronchodilator corticosteroid, beta-agonists, and anticholinergic inhalers), NRT and GSC were also provided.

### Nicotine replacement therapy (NRT)

Compared with the placebo or non-NRT control group, previous studies have indicated that NRT is a known effective method for smoking cessation^17-19^. Using nicotine cartridges (labelled 30 gums) containing 2 mg/mL of nicotine, NRT was administrated to the patients via transmucosal delivered nicotine polacrilex (nicotine gum) in an *ad lib* dose basis^10^, i.e. whenever the craving arose, the patient took a nicotine gum. Patients were taught how to use nicotine gums by the therapist.

### GSC treatment

The GSC model for treatment of alcohol-related problems was developed by Sobell et al.^12^, which has been evaluated by smokers and drug abusers. In GSC, the risks and barriers are assessed by the individual and suggestions for change are made, removing barriers instructions and including rewards. Participants receive personal feedback based on their evaluations and increased motivation. The findings^20^ indicate that if treatment is individualized, the motivation to change increases. This model was adopted based on CBT and motivational interview, consisting of one initial assessment session, four treatment sessions and two follow-up telephone calls. Participants were guided by the motivation enhancement principles and a self-help manual. All treatments in the three groups were delivered by the same therapist who was a trained CBT counsellor with over 15 years of experience in psychotherapy. This counsellor was trained to provide GSC treatment by a psychiatrist and a psychologist in a three-day workshop and subsequently treated five subjects before the study. The treatment sessions in the GSC study arm were tape-recorded in order to ensure treatment fidelity.

### GSC intervention protocol

The GSC intervention protocol consisted of five treatment sessions, including the consequences of smoking, deciding to change, discussing risky situations, identifying altered solutions to action, and planning for the future. In this study, GSC was applied in five 1-hour sessions for five weeks^21,22^.

### Outcomes

#### Primary and secondary outcomes

The primary outcome was smoking cessation rate, while the secondary outcomes included the rates of the following parameters: nicotine dependency, CAT, QoL, spirometry parameters, exhaled CO, and CO binding to haemoglobin, in patients over 29 weeks after treatment in the three groups.

### Statistical analysis

Normal distribution of data was evaluated using the Shapiro-Wilk test. Descriptive baseline values were presented as mean (±SD), median (inter-quartile range), or percentages. Chi-squared test or Fisher’s exact test were applied to compare the study groups in terms of categorical data. Comparisons of continuous data were performed using T-test or Mann-Whitney U test. An intention-to-treat analysis was conducted in order to assess the primary effects of the interventions on smoking cessation and pulmonary functions. A general linear model (GLM) of outcomes was developed for the study groups and compared using repeated measures of analysis of variance (ANOVA). The evaluation time and intervention state (GSC and NRT) were regarded as the within-subject and between-subject factors, respectively. The time groups (interaction terms) were considered as group differences (among three groups) in their response over time. The compound symmetry assumption was examined using Mauchly’s sphericity test.

Moreover, a generalised estimating equation (GEE) model was developed in order to control the potential confounders and compare the study groups in terms of the values of smoking cessation, pulmonary function, CAT, and QoL, at different points of time. It was also applied to determine the trend of changes after treatment. A p-value ≤0.05 was considered statistically significant. All analyses were performed using Stata 12 (Stata Corp, Texas, USA) and SPSS 16.0 (SPSS Inc., Chicago, IL, USA).

## RESULTS

### Participants

**Figure 1:**
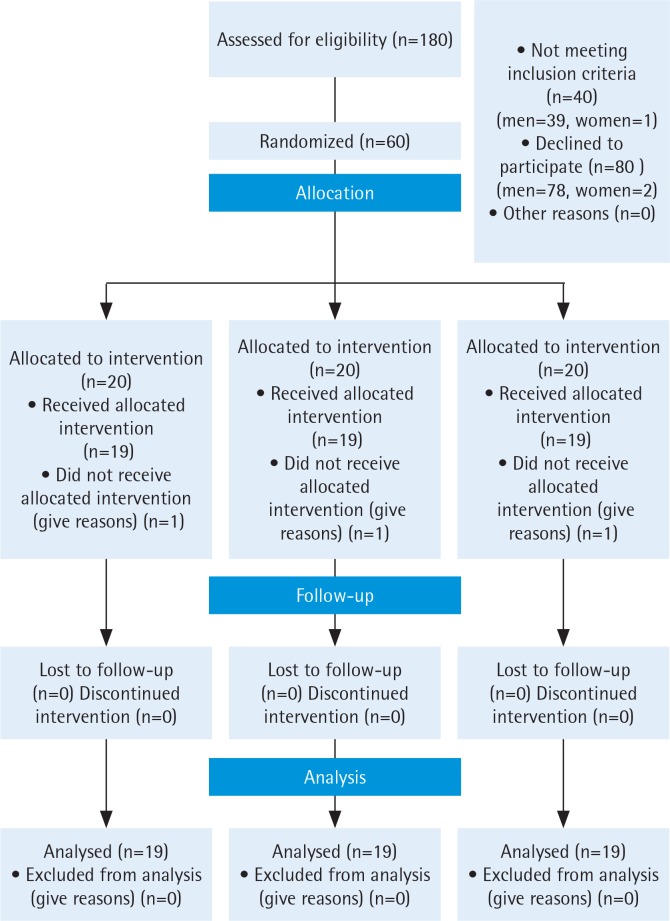
Flow diagram of patients’ randomization, intervention and analysis

This study screened a total of 180 patients with pulmonary medical records who were referred to the pulmonology clinic. However, only 60 patients were eligible, and since three subjects lost the follow-up processes, data from the other 57 patients were analyzed, and they were randomized into three groups (Figure 1). In this study, whenever the craving arose, the patient took nicotine gum, with an average consumption of 10 gums/day. The groups had no significant differences in mean age, marital status and other characteristics (occupation, motivation of quitting, importance of smoking cessation, smoker friends, craving, FEV1, FVC, FTND, and daily cigarette smoking) (Table 1).

**Table 1:** Demographic and clinical characteristics of patients in three groups

*Variable*	*Category*	*Group*			*p*
		*GSC (N=19)*	*NRT (N=19)*	*Combined (N=19)*	
**Age** (years) mean ± SD		50 ± 6	56 ± 10	54 ± 8	0.50
**Marital status** n (%)	Married	15 (31)	16 (32)	18 (37)	0.36
	Single/divorced/widowed	4 (50)	3 (38)	1 (13)	
**Employment** n (%)	Self-employed	14 (39)	11 (31)	11 (31)	0.51
	Employed	5 (24)	8 (38)	8 (38)	
**Motivation of quitting** n (%)	Desperate and unwilling	1 (50)	0	1 (50)	0.62
	Hopeful and very hopeful	18 (33)	19 (35)	18 (33)	
**Importance of smoking cessation** n (%)	Trivial and small	1 (50)	0	1 (50)	0.61
	Very much and too much	18 (33)	19 (35)	18 (33)	
**Smoker friends** n (%)	None of them and a few	13 (36)	13 (36)	10 (28)	0.50
	Half and most	6 (29)	6 (29)	9 (43)	
**Craving in TTM** mean ± SD		22 ± 8	23 ± 6	26 ± 8	0.21
**HSI** mean ± SD		1.8 ± 1	2 ± 1	1.7 ± 1	0.74
**FTND score** mean ± SD (>5 in 42.1% of patients)		4.7 ± 2	4.9 ± 3	4.9 ± 2	0.93
**Daily cigarettes** mean ± SD (range: 5–60, mean=23)		24 ± 13	26 ± 18	20 ± 7	0.71
**FEV1 act** mean ± SD		2.39 ± 0.57	1.94 ± 0.74	1.91 ± 0.73	0.62
**FVC act** mean ± SD		3.68 ± 0.71	3.18 ± 1.02	3.41 ± 0.83	0.82
**BMI** mean ± SD		27.41 ± 4.53	27.4 ± 3.79	23.93 ± 4.28	0.63
**CAT** mean ± SD		28.52 ± 6.76	34.68 ± 6.13	32.15 ± 6.95	0.36
**QoL** mean ± SD		31.26 ± 5.84	28.63 ± 7.58	29.15 ± 5.91	0.30

TTM: trans theoretical model, HSI: heaviness of smoking index, FTND: Fagerström test for nicotine dependence, FEV1: forced expiratory volume in the first second, FVC: forced vital capacity, BMI: body mass index, CAT: clinical assessment test, QoL: quality of life.

### Daily cigarette smoking and abstinence rate

**Figure 2:**
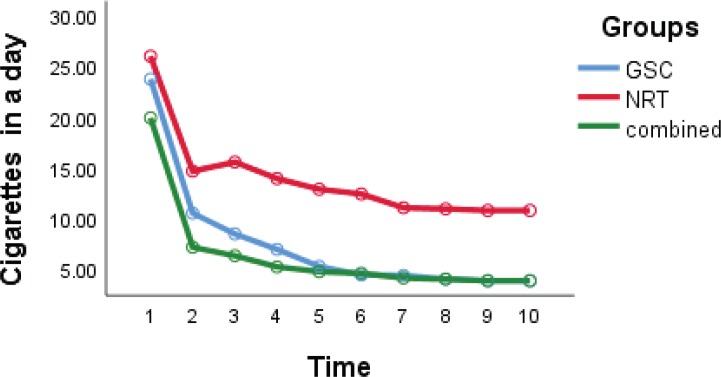
Daily cigarette trends over time in groups, at baseline and every 3 weeks

The daily number of cigarettes in the GSC group decreased from 24 to 4, in the NRT group from 26 to 11, and in the combined group from 20 to 6 (Table 2). As shown in Figure 2, the reduction of daily cigarette smoking in the GSC and the combined group was significantly larger than in the NRT group (interaction group effect) (p= 0.003). The abstinence rates in the NRT group over the 6, 12 and 29 weeks, were 5.3% (1 patient), 15.8% (3 patients) and 21.1% (4 patients), respectively; in the GSC group, the rates were 21.1% (4 patients), 31.6% (6 patients) and 47.4% (9 patients); and in the combined group, the rates were 36.8% (7 patients), 36.8% (7 patients) and 47.4% (9 patients), respectively. There was a statistically significant difference between the abstinence rate in the GSC or combined groups and the NRT group (p=0.001). The GEE model was adjusted for other variables that showed GSC reduced odds of smoking compared with the NRT group (interaction group effect) (OR=0.31; 95% CI: 0.022 – 0.545; p=0.001).

**Table 2:** Daily cigarettes, exhaled CO and CO Hb of participants, with scores at baseline and 3 weeks intervals after the interventions in three groups

	*Time*										*Between effect*	*Group effect*	*Interaction effect*
	*T1*	*T2*	*T3*	*T4*	*T5*	*T6*	*T7*	*T8*	*T9*	*T10*			
**Daily cigarettes**													
GSC	20 (16-30)	12 (1-18)	9 (1-15)	7 (1-10)	4 (0-10)	3 (0-9)	3 (0-9)	3 (0-9)	2 (0-9)	2 (0-9)	0.001	0.003	0.49
Nic	20 (12-30)	13 (6-20)	12 (8-20)	11 (7-20)	10 (5-18)	10 (4-17)	10 (4-16)	10 (3-16)	10 (3-16)	10 (3-16)	0.001		
Com	20 (15-30)	6 (0-14)	6 (0-12)	5 (0-10)	3 (0-8)	3 (0-8)	2 (0-8)	2 (0-7)	1 (0-7)	1 (0-7)	0.001		
**Exhaled CO**													
GSC	21 (13-38)	14 (8-25)	13 (7-20)	12 (6-15)	9 (6-14)	9 (6-14)	9 (6-14)	8 (5-12)	8 (3-12)	8 (3-12)	0.001	0.004	0.7
Nic	21 (16-31)	17 (12-23)	15 (11-17)	15 (10-18)	14 (10-17)	14 (10-16)	13 (7-16)	14 (7-16)	14 (7-16)	14 (7-16)	0.001		
Com	19 (14-27)	11 (7-20)	10 (6-13)	9 (3-11)	7 (3-10)	6 (3-10)	4 (3-10)	4 (3-9)	4 (3-9)	4 (3-9)	0.001		
CO HB*													
GSC	4 (2.7-6.8)	2.9 (1.9-4.6)	2.7 (1.8-3.8)	2.6 (1.6-3)	2.1 (1.6-2.9)	1.9 (1.6-2.9)	1.9 (1.6-2.9)	1.9 (1.4-2.6)	1.8 (1.1-2.4)	1.8 (1.1-2.4)	0.001	0.003	0.59
Nic	4 (3.2-5.6)	3.4 (2.6-4.3)	3.03 (2.4-3.4)	3 (2.2-3.5)	2.9 (2.2-3.4)	2.9 (2.2-3.2)	2.87 (1.8-3.2)	2.87 (1.8-3.2)	2.87 (1.75-3.2)	2.87 (1.8-3.2)	0.001		
Com	3.7 (2.9-5)	2.4 (1.8-3.8)	2.2 (1.6-2.7)	1.9 (1.1-2.39)	1.7 (1.1-2.2)	1.6 (1.1-2.2)	1.3 (1.1-2.2)	1.3 (1.1-2.07)	1.3 (1.1-2.07)	1.3 (1.1-2.07)	0.001		

* Carbone monoxide hemoglobin. Data are expressed as median (inter-quartile range).

### Exhaled carbon monoxide (CO)

The GEE model revealed that the exhaled CO and CO Hb reduction in the three study groups were statistically significant (time group effect) (p=0.004 and p=0.003, respectively). The exhaled CO reduction in the GSC (p=0.002) and combined groups (p=0.001) was lower than in the NRT group, and the reduction in the combined group was higher than in the GSC group (interaction group effect) (p=0.03). The CO Hb reduction in the GSC (p=0.003) and combined groups (p=0.001) was lower than that of the NRT group. The difference between this reduction in the combined and GSC groups was not statistically significant (interaction group effect) (p=0.24) (Table 2).

### Spirometry parameters

**Figure 3:**
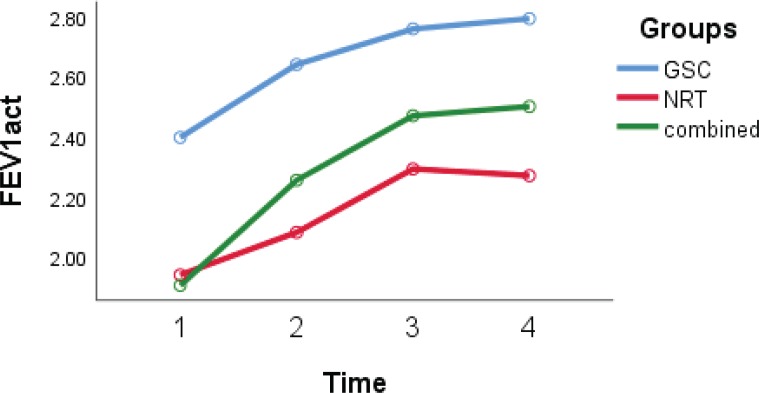
FEV1 trends over time in three groups, with scores at baseline, 12 and 29 weeks after treatment

**Figure 4:**
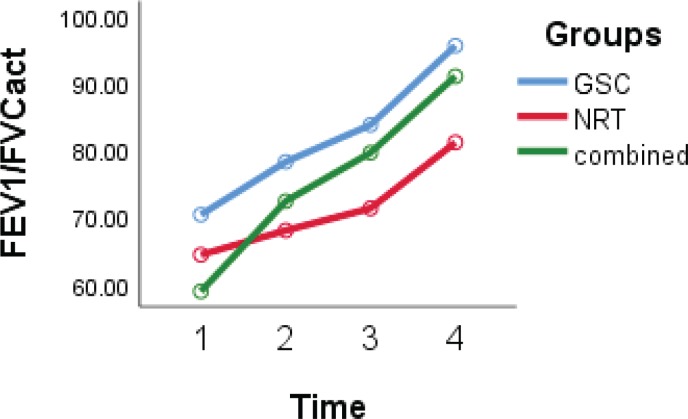
FEV1/FVC trends over time in three groups, with scores at baseline, 6, 12 and 29 weeks after treatment

According to the GEE model, differences in FVC and FEV1/FVC (Figures 3 and 4) in the three studied groups were statistically significant (interaction group effect) (p=0.05). The FVC and FEV1/FVC levels in the GSC (p=0.03 and p=0.04, respectively) and combined groups (p=0.04 and p=0.05, respectively) were higher than in the NRT group. The level of FEV1 in the NRT group was lower compared with the GSC group (-0.5; 95% CI: -0.9 – -0.12; p=0.009) and also lower in the combined group than in the GSC group (-0.38; 95% CI: -0.72 – -0.05; p=0.03) (interaction group effect).

### Nicotine dependence, clinical assessment test and quality of life

**Figure 5:**
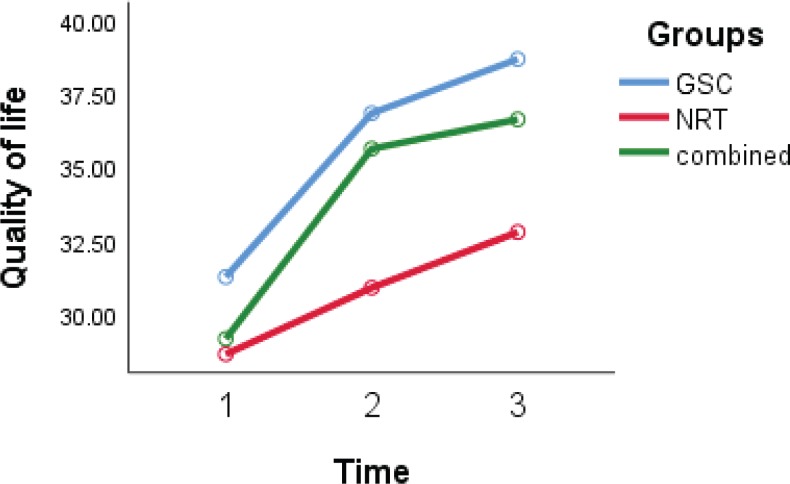
Quality-of-life trends over time in groups, with scores at baseline, 12 and 29 weeks after treatment

The GEE model revealed that the FTND was recovered in the three studied groups and it was lower in the GSC and combined groups in comparison to the NRT group, however, it was not statistically significant (p=0.1). CAT and QoL (Figure 5) were significantly recovered among the three groups (p=0.001 and p=0.04, respectively).

Clinical situation (measured by CAT) and quality of life (measured by QoL questionnaire) recovery in the GSC (p=0.002 and p=0.03, respectively) and the combined group (p=0.001 and p=0.004, respectively) (time group effect) were higher than in the NRT group. However, these differences in the combined and GSC groups were not statistically significant (p=0.24 and p=0.41, respectively). After adjustment for other variables, the GEE model revealed that the level of the CAT score in the NRT group was more than that of the GSC group (interaction group effect) (9.2; 95% CI: 5.0–13.4; p=0.001) and also the level of QoL score in the NRT group was lower than that of the GSC group (-4.5; 95% CI: -8.1 – -0.6; p=0.02). In other words, clinical situation and quality of life in the GSC was better than the NRT group.

## DISCUSSION

The 29-week follow-up of the GSC and combined therapy for 57 participants noted an increased smoking abstinence in comparison with the NRT group. The results of this study showed that cigarettes/day in GSC group decreased more than in the two other groups. Furthermore, smoking cessation rates in the GSC and combined groups were higher than those in the NRT group.

Several studies have been conducted on the effect of GSC as well as other psychological interventions for smoking/tobacco cessation. Also, many pharmaceutical therapies have been used in the treatment of addiction, including NRT for smoking cessation^23^. Sotoodeh Asl et al.^24^ reduced the smoking levels (65.4%) following individual short-term CBT. Moreover, an observational study has shown that 64.4% of people decreased the number of cigarettes by at least 50%, and 9.12% stopped smoking following behavioral and pharmaceutical therapies^25^, consistent with our and the Tan et al.^26^ study on reducing smoking and CO. Another study has shown that the amount of cessation at the time of hospital discharge was 55% for counseling with NRT, 43% for counselling without NRT, and 37% for a group with usual treatments. Moreover, the differences in quitting rates and QoL between counseling alone and routine treatment were not significant^11^. In contrast, in our study, these differences were significant among groups. In the present study, also 21% of the NRT group and 47% of the GSC group quit smoking during the 29-week of follow-up. Also, in a study in Hong Kong, abstinence rates at 4, 12, 26 and 52 weeks were all higher in the NRT + counselling group (35.8, 21.9, 16.8, and 20.1%) compared with the NRT group (28, 16.8, 11.2, and 14.3%). At 4 weeks, the combined group was more likely to quit smoking (OR=1.43, 95% CI: 1.00–2.05) than the NRT group. NRT + counselling group had a significantly higher abstinence rate (23.6%) than the NRT group (17.6%) at all time points. Combined NRT group was more likely to quit smoking (OR=1.43, 95% CI: 1.15–1.77)^27^. Other studies have reported an abstinent rate of 30.6% using NRT^28^, and 35% using counselling^29^.

Furthermore, numerous studies have been implemented for assessing cessation in adults with smoking-related diseases, such as COPD^30,31^. Research has indicated that a brief motivational interview accompanied by self-help material intervention was significantly more successful than usual treatment^32^. There is a necessity to compare different interventions, which may not necessarily yield the same results in different societies. In a randomized controlled trial performed in four psychiatric inpatient facilities in Australia, 745 participants were randomized to receive either usual care (n=375) or an intervention comprising a brief motivational interview and self-help materials (n=379) in the hospital, followed by a 4-month pharmacological and psychosocial intervention upon discharge. The primary outcomes assessed at 6 and 12 months post-discharge were 7-day point prevalence and 1-month prolonged smoking abstinence. At both 6 and 12 months post-discharge, the intervention group was significantly more likely to smoke fewer cigarettes/day, had a reduced cigarette consumption by ≥50% and experienced at least one quit attempt compared with the control group^33^. Accordingly, in our study, all of the smokers reduced their smoking. It should be noted that all received an intervention. Our findings are contrary to the findings of Molyneux et al.^11^ where the cessation rates of the NRT plus counselling group were higher than the NRT without counseling group. Lou et al.^34^ obtained an abstinence rate of 44.3% using behavioral intervention during 48 months. Sharifirad et al.^35^ showed a 46% stable cessation in the treatment group for two months via individual counseling; therapist skills affect the success rate. Hilberink et al.^36^ in a meta-analysis evaluated two counseling programs alone or in combination with NRT in smokers with COPD. Biochemically verified quit rates in comparison to the usual care resulted in a significantly higher self-reported success in smoking rate in the intervention group. It should be noted that all of the patients were in the preparation stage of the Trans Theoretical Model (TTM) of stages of change in our study. This preparation was probably influenced by the pulmonologist’s comments on the patient’s physical condition and on smoking cessation advice. Other studies have demonstrated that regardless of the intervention for smoking cessation, patients who recognized that their condition was due to cigarette smoking and had abnormal respiratory results were more prone to be in the preparation stage^7^. In our study, all patients had an airway obstruction and the effectiveness of GSC and GSC+NRT were equal in enhancing the quitting rate.

Furthermore, it is recommended to compare the effectiveness of GSC group with individual GSC. Camarelles et al.^37^, in a study in Spain, concluded that patients showed a lower compliance with group intervention than individual intervention. Moreover, individual smoking cessation interventions were not less effective than group interventions. In any case, the results of our study are consistent with the results of previous studies that evaluated the efficacy of individual psychological intervention for smoking cessation in Iran and other countries.

### Limitations

Our patients were all men, which can be considered as an important limitation. Moreover, the studied subjects were all in the preparation stage, which can result in different results compared with studying the participants in the other stages. A larger sample size with a longer follow-up may improve our knowledge regarding the effectiveness of smoking cessation programs. Furthermore, the stage of COPD may have impacted on study effectiveness, which we could not asses. Comparison of GSC with other well-known effective methods, such as bupropion or varenicline, should be examined in future studies. The efficiency of individual counseling for asymptomatic smokers remains uncertain and further nationwide multicenter investigations are required to investigate it in the future.

## CONCLUSIONS

Our participants were patients with COPD from the Mazandaran Province of Iran. The most significant result of this study was that GSC and combined GSC+NRT therapy were significantly more effective than NRT alone in promoting tobacco cessation. In addition, the findings indicate that the GSC and combined GSC+NRT are equally effective in smoking cessation, which indicates that the effectiveness of the combination method for smoking cessation in COPD patients can be attributed to the GSC. Health professions should emphasize using GSC for tobacco cessation as an integrated component of high-quality health care and promotion of QoL, spirometry parameters for all smokers, especially those with COPD. Besides, the culture of each society influences the effectiveness of different methods of psychotherapy, and the effective method in one society can not necessarily guarantee its effectiveness in another^38^. Finally, psychological treatments such as GSC are recommended to be performed in other societies and populations for smoking cessation.
