# Supplementary material for: Efficacy of guided self-change for smoking cessation in chronic obstructive pulmonary disease patients: A randomized controlled clinical trial
Source: Tob Induc Dis. 2019 Dec 11;17:90. doi: 10.18332/tid/114227 (PMC6915435; doi:10.18332/tid/114227)
Supplement: Supplementary file 1 [file TID-17-90-s1.pdf]

PROTOCOL

Guided self-change(GSC) is a shortened cognitive behavioral motivational intervention that was first designed to help alcohol consumers to aid them to identify and to utilize individual abilities to solve their dependency problem (1).This culture-sensitive treatment is applied to variety of individuals, couples or groups(2).The individual form of this treatment is standard and applicable for clinical and research applications (3).Its effectiveness in reducing alcohol consumption is 53.8% and the cessation rate is significant after one post-treatment year (4).

The rate of smoking cessation is 44% in a natural way and it is 55% and in GSC is 35%(5) . Patients undergoing GSC allocate less time to learning and training than cognitive-behavioral therapy since they are presented in their self-treatment booklet. Moreover, they are encouraged to reading the booklet during the treatment sessions and help them control problems such as low motivation, depression or acute crying. At the end of the treatment, the therapist and the patient will review the treatment process and discuss the future treatment(5) .GSC treatment for substance abuse combines cognitive-behavioral techniques, motivational interviewing, and relapse prevention aimed to help clients evaluate their substance and alcohol-related problems, and improve their own choices for change. This method can be individual or group-specific, and it is specifically designed for people who are not severely affected by their illness and substance problems (6) .

Considering the research and socio-cognitive learning theory that states that people are more involved in the goals they determine themselves, we decided to provide suggestions for them rather than setting goals to give them some information to make a responsible decision for them. Moreover, the clients are asked to provide a functional analysis of their high-risk situations and their action plans and change options. A functional analysis is performed through reading and completing homework that they discuss with their therapist during the sessions(8 ,7) .

Since GSC treatment and nicotine replacement therapy are both effective in treating smoking problems, moreover, smoking cessation improves the pulmonary function of patients with chronic obstructive pulmonary disease, on the other hand, GSC is culture-sensitive and it was used in no research in Iran so far, the researchers decided to investigate the effectiveness of adding GSC to NRT for smoking cessation in patients with COPD in a randomized, clinical trial with the control group in Imam Khomeini Hospital in Sari, Iran.

Design and Methodology

The present study is a clinical of pre-test post-test trial, with control group, aimed to investigating the effectiveness of GSC treatment in smoking cessation and on the performance of pulmonary function (improvement of pulmonary test status) in patients with COPD in the Research Center for Psychiatry and Behavioral Sciences of the Institute of Addiction and Lung Research Center of Imam Khomeini Hospital in Sari, Iran, associated to Mazandaran University of Medical Sciences in 2016. The trial protocol was registered at the Iranian Clinical Trials

Registry (IRCT201609271457N11; www.irct.ir)and performed in accordance with the Declaration of Helsinki and its subsequent revisions. Participants were said study information, and consent forms to sign and return. Patients were informed of their right to withdraw from the trial at any time. The study was performed between December 2016 to November 2017. The statistical population consisted of all COPD patients referred to the pulmonary clinic of Imam Khomeini Hospital.

Inclusion criteria

Inclusion criteria include age over 45, chronic obstructive pulmonary disease and nicotine dependency. Who had persistent airway obstruction with $FEV_1/FVC < 70\%$ and $FEV_1 < 90\%$ of predicted normal value; and were willing to follow the study protocol that diagnose and refer by a pulmonologist.

Exclusion criteria

Exclusion criteria include other systemic diseases such as diabetes, normal primary spirometry, respiratory failure, and Contraindications for nicotine gum consumption (sensitivity, recent heart attacks, dangerous arrhythmias, severe angina, hyperthyroidism, insulin-dependent diabetes, active peptic ulcers, pregnancy, lactation, and children), severe psychiatric disorders such as psychosis, severe depression and anxiety.

Randomization and matching

All of the participants were assigned to either the intervention group, matched in age, education and nicotine dependency in the three groups. Block randomization with a block size of four was used for the allocation. The randomization was conducted via a SPSS software randomization system by an independent investigator with no contact with the participants or researchers. The number of participants and the type of intervention were packed in a closed packet and then, by visiting the patient and after an initial assessment for inclusion criteria, was opened.

Blinding

Participants and the clinical practitioner were blinded in the initial assessment for inclusion criteria, but, neither participants nor the clinical practitioner were blinded during the clinical trial. It was not feasible to mask participants to allocation to GSC or gum or combine.

Sampling

Based on the previous study (Sotoudeh Asl et al.) and considering the mean and standard deviation of the difference between the reduction in the number of cigarettes equal to 1 and 0. 8 cigarettes after GSC intervention, moreover, considering the power of study equal to 80% and the probability of the first type error equal to 0. 01, the sample size is 15 participants in each group (9),Furthermore, according to the study of Sharifirad et al. and considering the ratio of smoking stopped people in the intervention and non-intervention groups equal to 46% and 4%, and considering the power of study equal to 80% and the probability of the first type error equal to 0.05, respectively, the sample size is calculated as 15 in each group (10) .

Accordingly, three intervention groups will be created; The "GSC" group, the "NRT" group, and the group of "combine of GSC and NRT". Each group includes 15 people,however, increasing the power of study, we assigned 20 participants in each groups that one patients in each group, discontinued the study, after allocation.

Procedures

Nicotine cartridges (labelled 30gums) contained 2 mg nicotine per mL. The first, participants randomized to the nicotine and combine group were invited to take part in testing, and completed the testing regimen. We chose nicotine gums (Nicolife) because they are the most popular NRT product in Iran, have proven effectiveness, and few known adverse events. All randomized participants were referred to Mostafavian clinic in Imam Khomeini hospital in sari city. After randomization, additional baseline data were collected e.g. education, smoking and quitting history, medication, Nicotine dependency and other related data

The primary outcome was continuous smoking abstinence (self-reported abstinence over the whole follow-up period), 6 months after quit day, verified at that point in time by exhaled breath carbon monoxide measurement, using Bedfont Micro Smokerlyzers (Bedfont Scientific, Maidstone, UK). Secondary outcomes assessed at 1.5, 3, and 6 months post quit day were: continuous abstinence, number of tobacco cigarettes smoked per day, withdrawal symptoms, smoking latency, and adverse events.

First, the purpose and method of research and the spirometry side effects were described for patients. The patients was completed a consent form. All patients completed the questionnaires of personal information, nicotine dependency (Fagerstrom in the self-rating state with controlling and guidance of practitioner, before the beginning of the intervention. The primary spirometry was taken in the Mostafaean Clinic and the carbon monoxide was measured in exhalation air of the patients. **All evaluations (spirometry and exhalation carbon monoxide measurement) were performed in a blind form**.

Then, in addition to common treatments (inhaler bronchodilator, spray containing inhaler corticosteroid, inhaler beta-agonist and inhaler anticholinergic), NRT and GSC were performed directed by a psychotherapist trained in 5 individual sessions hour for 5 weeks (and, if necessary, additional treatment sessions at the request of the patient) in Imam Khomeini Hospital for the experimental groups. The reason for choosing individual psychotherapy is the effectiveness of this method compared to the group therapy (22, 47). In this study, there was no restriction on the nicotine consumption (fixed regimen) .(11)

After the intervention, the patients were again asked to complete the questionnaire, the spirometry was repeated every 45 days after the onset of the treatment and 6 months follow up and 4 spirometry test were performed for each patient. Carbon monoxide of the exhaled air was also measured simultaneously with spirometry and during follow up as 10 times for each patients.

Questionnaires

Individual information questionnaire includes age, gender, martial status, occupation and education.

Trans theoretical Model (TTM)

Based on this pattern, a questionnaire or tool was developed to determine the change level of the patients and the motivation level to quit smoking. Moreover, the questionnaire was based on the structures of the model of Stage of Change, used by Dickelmante and Prochaska including 5 questions based on the change stages (pre-contemplation, contemplation, preparation, act, and maintenance) (12).Validity and reliability of this study were confirmed in the Sharifirad study in Iran (α= 0.8) .(10)

Fagerstrom Test for Nicotine Dependency (FTND)

The questionnaire used to assess the severity of nicotine dependency includes 6 questions regarding the time of use, the number of cigarette, the best cigarettes, early use during the day, consumption during illness and in prohibited places. The first two questions include 4 answers and the next 4 questions involve 2 answers. The score of 4-response questions is 3, 2, 1 and 0, respectively, and the score of 2-response questions is 1 and 0, which include a total of zero to 10 points. Scores 0 to 4 are considered as weak dependence, the score between 5 and 7 as moderate dependence and the scores of 8 to 10 are considered as severe dependencies (13) .In several studies, the validity and reliability of this test were confirmed (13, 14). The reduction of Cronbach's alpha coefficient in one study was reported as 0.56 (15)and in another study was reported as 0.68 (16).Moreover, its correlation with the Autonomy over Tobacco Scale (AUTOS) was 0.63-0.72 (17). Cronbach's alpha coefficient value of Persian version for 230 smokers was obtained as 0.71 and the validity was 0.63 (18) .The reduction of Cronbach's alpha coefficient value in the test was calculated as 0.71 in another study in Iran (10).

The heaviness of smoking index (HSI)

The first two FTCD items make up the HSI, scored 0–6(19)

Pulmonary function assessment

The pulmonary function is evaluated based on the results of spirometry (FEV1, FVC) by the spirometric device and the exhaled carbon monoxide is examined by Smokerlyzer.

Spirometry

Spirometry is performed and interpreted based on the guidelines of the American Thoracic Association. Spirometry is the most frequently used pulmonary function test and enables health professionals to make an objective measurement of airflow obstruction and assess the degree to which it is reversible. As a diagnostic test for COPD, spirometry is a reliable, simple, non-invasive, safe, and non-expensive procedure(20). Different thresholds of FEV_1 and dyspnea are used in different staging systems and with different guidelines (21, 22).COPD with a FEV1/

FVC ratio (FEV%) in men is less than 88% and in women is less than 89%. Moreover, the FEV of 70 and higher is considered as the moderate, within 50-69 as the less than moderate and the value of less than 50 is considered as the severe (23). Among smokers with COPD, stopping smoking reduces the rate of decline in forced expiratory volume in 1 s(FEV1), but lost lung function is not restored(24). the new GOLD document preserves its previous cutoffs for FEV_1 (mild≥80%, moderate 50–79%, severe 30–49%, and very severe <30%), together with a combined COPD risk assessment evaluated with previous spirometric classification divided into groups (FEV_1%≥50% and ≤49%) along with the individual patient history of exacerbations in the preceding year (0–1 and ≥2 or 1 severe exacerbation) (25).

Exhaled Carbon Monoxide Measurement

Exhaled CO (eCO) assessment of lung is a simple, noninvasive tool(26).who smoked an average of ten or more cigarettes per day during the previous year, had an exhaled carbon monoxide concentration greater than 10 parts per million (ppm) at screening(27).The amount of exhaled carbon monoxide is measured by Smokerlyzer . The concentration of the lost carbon monoxide is measured to confirm the reduced smoking or smoking cessation, in PPM. Concentrations of up to 10 indicate that the participants are smokers the concentration of 6-10 represents an sporadic smoking (sometimes) and the concentration of less than 6 indicates non-smoking (28, 29).Since the determination of exhaled carbon monoxide is non-invasive, inexpensive and immediate results-based method, it is a selective approach to clinical research (30).The half-life of carbon monoxide is 3-6 hours, which is dependent on exercise and environmental carbon dioxide. Previous studies showed that smoking in the last 24 hours leads to an increase in the level of respiratory monoxide to a greater than normal physiological level. However, this can be both dependents on the number of cigarettes and the last smoking (31, 32)

GSC intervention protocol:

Session 1: screening and evaluation

The questionnaire of demographic information, history of smoking, the first smoking, and the amount previous smoking were collected for the present and the past. The referrals' reasons for the change were evaluated (considering the questionnaire of TTM). The profit and loss analysis was performed and the questionnaires of Fagerstrom, quality of life, and CAT test were completed. The goal of patient about consumption change was determine. The homework involves completing the daily smoking form for different conditions and its related emotions and completing the cast-benefit table for quiting or non-quitting of smoking.

Session 2: Deciding to change

The completed form of the smoking in the previous week was discussed, the goals and treatment logic were reviewed, and the reasons for the change were discussed. Detecting the smoking stimulants and high-risk situations and their problematic consequences were explained and the patients were asked to complete the form for the daily consumption in a next week at home.

Session 3: Discussing Risky Situations

The completed smoking cessation form for the previous week was discussed, the high-risk situations were addressed and the operational solutions were sought from the patients. Homework involves identifying consumption situations during the future week, and coping with them, as well as completing the daily smoking form.

Session Four: Identify various solutions to action

The completed consumption form of the previous week was discussed aimed to identify the beneficial methods in high-risk situations. Redetermination the goal of smoking was done by the patients. As the homework, the patients were asked to monitor their consumption status, and identify new consumption situations and complete the daily consumption form.

Session 5: Steps to the Future

The completed form of the previous week consumption and the patients' progress were reviewed. The patients were asked to assess their commitment, consider the cast and benefits from changes, and their progress was encouraged. Phone follow-ups were performed if necessary (33) .

Adverse events with NRT

8 participants that take nicotine had weight gain over 6 kg,1 patient had gingival ulcer,2 patients had dyspnea,2 patients had insomnia,2 patients had restlessness,3 participants had hearth burn,1 patient had headach,1 patient reported crying,1 patient reported sexual impotency and 2 patients had coughing. The patient with gingival ulcer, hadn't any teeth and with dentist consulting was treated. The patient that reported sexual impotency decline nicotine consumption and reported had Improved. The patient that reported crying referred to psychiatrist, the patient had history of depression almost 3 years ago. Participants with over weight gain refer to a nutritionist. Others adopted with their complaint. All of them continued their smoking cessation treatments. Nicotine withdrawal severity appears multi determined, related to cigarette dependence, demographic variables, and other substance use (34).

Dropping in allocation

We had 1 patient in each group that hadn't participated in interventions after allocation. The first was a 54-year-old man with free job and diploma, with alcohol and opiate consumption who smoked two cigarette packs a day that allocated in the GSC group. He believed that GSC did not solve his smoking problem.

The other was a 67-year-old man with a primary education and retired, who smoked two cigarette packs a day and allocated in the nicotine group, he believed that the use of nicotine in the past, had increased his craving for cigarette smoking.

The third was a 60-year-old man assigned in combine group who smoked 1.5 cigarette packs a day ,diploma and retired that believed he needed a different drug that hate him from smoking.

References

1. Saladin ME, Santa Ana EJ. Controlled drinking: More than just a controversy. Current Opinion in Psychiatry. 2004;17(3):175-87.
2. Gil AG, Wagner EF, Tubman JG. Culturally sensitive substance abuse intervention for Hispanic and African American adolescents: Empirical examples from the Alcohol Treatment Targeting Adolescents in Need (ATTAIN) Project. Addiction (Abingdon, England). 2004;99(s2):140-50.
3. Breslin C, Li S, Sdao-Jarvie K, Tupker E, Ittig-Deland V. Brief treatment for young substance abusers: a pilot study in an addiction treatment setting. Psychology of Addictive behaviors. 2002;16(1):10.
4. Tubman JG, Wagner EF, Gil AG, Pate KN. Brief motivational intervention for substance-abusing delinquent adolescents: Guided self-change as a social work practice innovation. Health & social work. 2002;27(3):208.
5. Sobell MB, Sobell LC. Guided self-change model of treatment for substance use disorders. Journal of Cognitive Psychotherapy. 2005;19(3):199-210.
6. Sobell LC, Sobell MB, Leo GI, Agrawal S, Johnson-Young L, Cunningham JA. Promoting Self-Change With Alcohol Abusers: A Community-Level Mail Intervention Based on Natural Recovery Studies. Alcoholism: Clinical and Experimental Research. 2002;26(6):936-48.
7. Stratelis G, Mölstad S, Jakobsson P, Zetterström O. The impact of repeated spirometry and smoking cessation advice on smokers with mild COPD. Scandinavian journal of primary health care. 2006;24(3):133-9.
8. Chen J, Chen Y, Chen P, Liu Z, Luo H, Cai S. Effectiveness of individual counseling for smoking cessation in smokers with chronic obstructive pulmonary disease and asymptomatic smokers. Experimental and therapeutic medicine. 2014;7(3):716-20.
9. Sotoodeh Asl N, Taher Neshatdost H, Kalantari M, Talebi H, Mehrabi HA, Khosravi AR. The effectiveness of cognitive behavioral therapy on the reduction of Tobacco Dependency in patients with essential hypertension. Journal of Research in Behavioural Sciences. 2011;9(2).
10. Sharifirad GR, Eslami AA, Charkazi A, Mostafavi F, Shahnazi H. The effect of individual counseling, line follow-up, and free nicotine replacement therapy on smoking cessation in the samples of Iranian smokers: Examination of transtheoretical model. Journal of research in medical sciences: the official journal of Isfahan University of Medical Sciences. 2012;17(12):1128.
11. Killen. Evaluation of a treatment approach combining nicotine gum with self-guided behavioral treatments for smoking relapse prevention. Journal of consulting and clinical psychology. 1990;58(1):85-92.
12. Velicer WF, DiClemente CC, Prochaska JO, Brandenburg N. Decisional balance measure for assessing and predicting smoking status. Journal of personality and social psychology. 1985;48(5):1279.
13. Heydari GR, Ariyanpour M, Kashani BS, Ramezankhani A, Tafti SF, Hosseini M, et al. Tobacco dependency evaluation with fagerstrom test among the entrants of smoking cessation clinic. Tanaffos. 2007;6(4):47-52.
14. Masjedi MR, Azaripour Masooleh H, Hosseini M, Heydari GR. Effective factors on smoking cessation among the smokers in the first “smoking cessation clinic” in Iran. Tanaffos. 2002;1(4):61-7.
15. Uysal MA, Kadakal F, Karsidag C, Bayram NG, Uysal O, Yilmaz V. Fagerstrom test for nicotine dependence: reliability in a Turkish sample and factor analysis. Tuberk Toraks. 2004;52(2):115-21.

16. Kassim S, Salam M, Croucher R. Validity and reliability of the fagerstrom test for cigarette dependence in a sample of Arabic speaking UK-resident Yemeni khat chewers. Asian Pacific journal of cancer prevention. 2012;13(4):1285-8.
17. DiFranza JR, Wellman RJ, Savageau JA, Beccia A, Ursprung W, McMillen R. What Aspect of Dependence Does the Fagerström Test for Nicotine Dependence Measure? ISRN Addiction. 2012;2013.
18. Sarbandi F, Niknami S, Hidarnia A, Hajizadeh E, Masooleh HA, Nobari SE. Psychometric properties of the Iranian version of the fagerstrom test for nicotine dependence and of heaviness of smoking index. Journal of Research & Health. 2015;5(1):96-103.
19. Riaz M, Lewis S, Coleman T, Aveyard P. Which measures of cigarette dependence are predictors of smoking cessation during pregnancy? Analysis of data from a randomized controlled trial. 2016;111(9):1656-65.
20. Soriano JB, Zielinski J, Price D. Screening for and early detection of chronic obstructive pulmonary disease. The Lancet. 2009;374(9691):721-32.
21. BTS guidelines for the management of chronic obstructive pulmonary disease. The COPD Guidelines Group of the Standards of Care Committee of the BTS. Thorax. 1997;52 Suppl 5:S1-28.
22. Miravitlles M S-CJ, Calle M, Molina J, Almagro P, et al. (2012) Pharmacological treatment of stable COPD. Arch Bronconeumol 48: 247–257.
23. Reda AA, Kotz D, Kocks JW, Wesseling G, van Schayck CP. Reliability and validity of the clinical COPD questionniare and chronic respiratory questionnaire. Respiratory medicine. 2010;104(11):1675-82.
24. Rigotti NA. Smoking cessation in patients with respiratory disease: existing treatments and future directions. The Lancet Respiratory Medicine.1(3):241-50.
25. Vestbo J, Hurd SS, Agusti AG, Jones PW, Vogelmeier C, Anzueto A, et al. Global strategy for the diagnosis, management, and prevention of chronic obstructive pulmonary disease: GOLD executive summary. American journal of respiratory and critical care medicine. 2013;187(4):347-65.
26. Zarghami M, Taghizadeh F, Sharifpoor A. Role of Fireplaces in the Measurement of Exhaled Carbon Monoxide in Two Smokers with Chronic Obstructive Pulmonary Disease. Case Reports in Clinical Practice. 2017;2(2):46-9.
27. Anthenelli RM, Benowitz NL, West R, St Aubin L, McRae T, Lawrence D, et al. Neuropsychiatric safety and efficacy of varenicline, bupropion, and nicotine patch in smokers with and without psychiatric disorders (EAGLES): a double-blind, randomised, placebo-controlled clinical trial. Lancet (London, England). 2016;387(10037):2507-20.
28. Cropsey KL, Jackson DO, Hale GJ, Carpenter MJ, Stitzer ML. Impact of self-initiated pre-quit smoking reduction on cessation rates: results of a clinical trial of smoking cessation among female prisoners. Addictive behaviors. 2011;36(1):73-8.
29. Korte KJ, Capron DW, Zvolensky M, Schmidt NB. The Fagerström Test for Nicotine Dependence: Do revisions in the item scoring enhance the psychometric properties? Addictive behaviors. 2013;38(3):1757-63.
30. Javors MA, Hatch JP, Lamb RJ. Cut-off levels for breath carbon monoxide as a marker for cigarette smoking. Addiction (Abingdon, England). 2005;100(2):159-67.
31. Benowitz NL, Jacob P, Hall S, Tsoh J, Ahijevych K, Jarvis M, et al. Biochemical verification of tobacco use and cessation. Nicotine and Tobacco Research. 2002;4(2):149-59.
32. Kauffman RM, Ferketich AK, Murray DM, Bellair PE, Wewers ME. Measuring tobacco use in a prison population. Nicotine & Tobacco Research. 2010:ntq048.
33. Sobell MB LC. Guided self - change for employee assistance programs: counsellor's manual, c1998. 1998:63-7.
34. Soyster P, Anzai NE, Fromont SC, Prochaska JJ. Correlates of nicotine withdrawal severity in smokers during a smoke-free psychiatric hospitalization. Prev Med. 2016;92:176-82.

Interpretation

In our study, GSC, with or without NRT, was modestly effective in helping smokers to quit. GSC was more effective than or had similar effectiveness as combined treatment; however, more rigorous studies regarding comparing the effectiveness of NRT, GSC, and combined treatment are needed in smoking cessation in COPD patients in other populations and cultures. NRT was associated with a few adverse effects, but the smokers adapted to them in longer-term in our study. Elderly COPD patients with a long history of smoking and low education need to be taught how to overcome temptation to smoke in a variety of risk situations. We believe our study provides a benchmark for the application and effectiveness of GSC against which to design future more rigorous trials. Furthermore, since it has higher acceptability (as shown by the present study) among smokers than NRT and seems to have no greater risk of adverse effects, GSC also has the potential for enhancing public health. On the other hand, GSC is specifically designed for people who are not severely affected by their illness and substance abuse [1]. The patients in our study found a correlation between their smoking and COPD condition that can enhance their motivation to quit smoking. Improved cessation rates were found in COPD smokers, many of them suffering from a cough and dyspnea, widely considered to be hazardous effects of smoking. Secondly, low spirometry results, low exhaled CO, and a COPD diagnosis also contribute to motivating smokers to cut down on or quit smoking.

1. Sobell LC, Sobell MB, Leo GI, Agrawal S, Johnson-Young L, Cunningham JA. Promoting Self-Change With Alcohol Abusers: A Community-Level Mail Intervention Based on Natural Recovery Studies. *Alcoholism: Clinical and Experimental Research.* 2002;26(6):936-948.

Other findings

The age of smoking onset was 8-34 (mean=19.6) years, and the duration of smoking was 9-59 (mean=32.9) years. The number of cigarettes smoking per day was 5-60 (mean=23). Moreover, the level of nicotine dependence (FTND) was more than 5 in 42.1% of patients, and in 57.9% of patients, it was less than or equal to 5 with mean 4.8. Moreover, 53 patients (93%) smoked after a main meal and 13 (8.22%) smoked after sex regularly. Among them, 16 (28.1%) smoked opium, 5 (8.8%) used methadone, 4 (7%) used alcohol, and 6 (10.5%) used alcohol and smoked opium with cigarettes. A total of 38 patients (67%) had a history of smoking in their fathers or brothers. Cutoff values for staging based on FEV1%pred were those proposed by GOLD (namely 80%, 50%, and 30%) and quartiles of FEV1%pred[1]. According to this categorization, 4

(7%) of the patients had very severe COPD, 11(19.3%) of them had severe COPD, 35(61.4%) of the patients had moderate COPD, and 7(12.3%) of the patients had mild COPD. Self-reported smoking declined steadily over the 6, 12 and 29 weeks periods, from 23.2 (±1.7) to 7.6 (±1.0) and 6(±1.0) cigarettes/day respectively (P<0.001). The mean exhaled carbon monoxide decreased from 22.6 (±1.5) to 12.8 (±0.9) and 8.6(±0.7) ppm after 6, 12 and 29 weeks (P<0.001). Also, HSI and non-HSI exhibited similar results to the full FTND

Huang TH, Hsiue TR, Lin SH, Liao XM, Su PL, Chen CZ. Comparison of different staging methods for COPD in predicting outcomes. Eur Respir J. 2018;51(3)

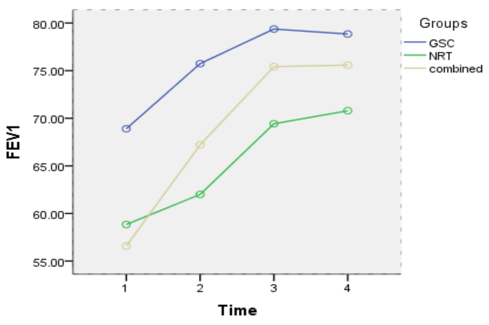

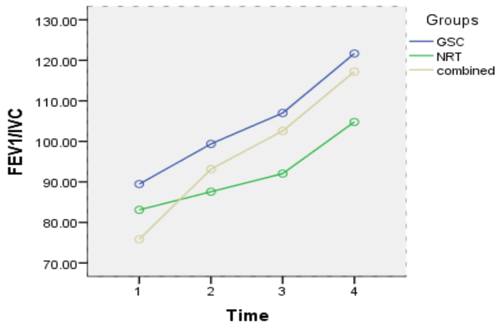

Figure 2: FEV1 trends over the time in groups   **Figure 3**: FEV1/IVC trends over the time in groups

Spirometry parameters

Forced vital capacity (FVC- act) and FEV1/IVC act in three groups of study were statistically significant (group effect, P=0.05) (table 2). Contrasts revealed that FVC- act and FEV1/IVC- act levels in GSC (P=0.03 and P=0.04 respectively) and combined group (P=0.04 and P=0.05 respectively) was higher than NRT group.After adjusting of other variables, GEE model revealed that the FVC-act level of the NRT group was lower than the GSC group (-0.52, 95% CI: -1- -0.02, P=0.04) and FVC-Pred level in the NRT group was lower than the GSC group (-0.52, 95% CI: -1- -0.02, P=0.04). The level of FEV_1-act in the NRT group was lower than the GSC group (-0.5, 95% CI: -0.9- -0.12, P=0.009) and furthermore, this variable was also lower in the combined group than in the GSC group (-0.38, 95% CI: -0.72- -0.05, P=0.03). The FEV_1-act/pred level in the NRT group was lower than the GSC group (-9.7, 95% CI: -17.9- -1.5, P=0.02). The level of FEV1/IVC-Pred in the NRT group was lower than the GSC group (-4.3, 95% CI: -7.8- -0.81, P=0.02) and the level of FEV1/IVC-act in the NRT group was lower than the GSC (-5.9, 95% CI: -10.4-(-1.5), P=0.03). Furthermore, this variable was less in the combined group than in the GSC group (-5.9, 95% CI: -10.4-(-1.5), P=0.03). The level of FEV1/IVC actPred in the NRT

group was lower than the GSC group (-6, 95% CI: -10.4-(-1.5), P=0.008) and furthermore this variable was also lower in the combined group than in the GSC group (-6, 95% CI: -11.3-(-0.5), PV=0.03) (table 2). Also,The relationship between MMRC with FEV1act/pred, FVCact/pred was significant (p=0.001).

© 2019 Zarghami M. et al.
